# Sensitivity of Quantitative Signal Detection in Regards to Pharmacological Neuroenhancement

**DOI:** 10.3390/ijms18010101

**Published:** 2017-01-05

**Authors:** Maximilian Gahr, Bernhard J. Connemann, Carlos Schönfeldt-Lecuona, René Zeiss

**Affiliations:** Department of Psychiatry and Psychotherapy III, University of Ulm, Leimgrubenweg 12-14, 89075 Ulm, Germany; bernhard.connemann@uni-ulm.de (B.J.C.); carlos.schoenfeldt@uni-ulm.de (C.S.-L.); rene.zeiss@uni-ulm.de (R.Z.)

**Keywords:** addictovigilance, atomoxetine, brain doping, methylphenidate, modafinil

## Abstract

Pharmacological neuroenhancement (PNE) is a form of abuse and has not yet been addressed by methods of pharmacovigilance. In the present study, we tested if quantitative signal detection may be sensitive in regards to PNE. We evaluated the risk of drug abuse and dependence (DAAD) related to substances that are known to be used for PNE and divided this group into agents with (methylphenidate) and without a known abuse potential outside the field of PNE (atomoxetine, modafinil, acetylcholine esterase inhibitors, and memantine). Reporting odds ratios (RORs) were calculated using a case/non-case approach based on global and country-specific drug safety data from the Uppsala Monitoring Centre (UMC). Both control substances (diazepam and lorazepam) and methylphenidate were statistically associated with DAAD in all datasets (except methylphenidate in Italy). Modafinil was associated with DAAD in the total dataset (ROR, 2.7 (95% confidence interval (CI), 2.2–3.3)), Germany (ROR, 4.6 (95% CI, 1.8–11.5)), and the USA (ROR, 2.0 (95% CI, 1.6–2.5)). Atomoxetine was associated with DAAD in the total dataset (ROR, 1.3 (95% CI, 1.2–1.5)) and in the UK (ROR, 3.3 (95% CI, 1.8–6.1)). Apart from memantine, which was associated with DAAD in Germany (ROR, 1.8 (95% CI, 1.0–3.2)), no other antidementia drug was associated with DAAD. Quantitative signal detection is suitable to detect agents with a risk for DAAD. Its sensitivity regarding PNE is limited, although atomoxetine and modafinil, which do not have a known abuse potential outside PNE, and no antidementia drugs, whose use in PNE is presumably low, were associated with DAAD in our analysis.

## 1. Introduction

Pharmacological neuroenhancement (PNE) is defined as the voluntary use of psychoactive substances over-the-counter (OTC), by prescription or through illicit drugs to enhance the function of certain cognitive domains (e.g., attention, concentration, motivation, vigilance, and working memory) by healthy persons without a medical indication [1]. Several epidemiological studies suggest that PNE is a common practice in several Western countries [2,3], is widespread among students [2,3,4,5], and can be found in particular work environments such as science [6] and finance fields [7]. Currently, PNE is subject of a controversial debate that addresses medical, legal and ethical aspects and receives increasing public attention [1,8,9]. Pharmaceutical agents frequently used for PNE are psychostimulants (e.g., amphetamines, methylphenidate), atomoxetine, modafinil, and antidementia drugs (e.g., acetlycholine esterase inhibitors, memantine) [3,10,11,12]. It has not yet been sufficiently studied regarding which substances are used most frequently for PNE. However, antidementia drugs seem to be less frequently used for PNE; in contrast, atomoxetine, methylphenidate and modafinil may be used more frequently for PNE [3]. Controlled clinical studies indicate positive effects of modafinil and methylphenidate on certain cognitive domains in healthy persons [13,14], whereas the results of clinical studies regarding the cognitive enhancing effects of acetylcholine esterase inhibitors and memantine in healthy persons are conflicting [15]. Cognitive enhancing effects of atomoxetine have not been studied sufficiently; however, some evidence suggests that atomoxetine may improve error monitoring/sensitivity and inhibitory control in healthy persons [16].

Although research concerning the long-term outcome of individuals who perform PNE is currently absent, there is some evidence suggesting that PNE may lead to substance use disorders [17]. In addition, from a regulatory and medical perspective, PNE should be regarded as a form of abuse. Monitoring the potential for abuse and dependence of psychoactive substances falls within the scope of pharmacovigilance (“addictovigilance”) [18,19]. Addictive behaviours related to pharmaceutical agents are possible adverse drug reactions (ADRs). In this respect, addictive behaviours related to certain substances may be reported to spontaneous reporting systems [20]. Methods of pharmacovigilance such as quantitative signal detection have been repeatedly applied successfully to detect the abuse potential of pharmaceutical agents [19,21,22]. PNE as a form of abuse has not yet been addressed by methods of pharmacovigilance. In this regard, however, it is necessary to consider that data mining methods (e.g., quantitative signal detection) that are frequently used in databases of spontaneous reporting systems of ADR [23] do not allow for including case-sensitive data, e.g., concerning the individual motive for (ab)using a particular substance or the presence of comorbid substance use disorders. This implicates that the ADR reports analyzed by quantitative signal detection are not specific with regard to the underlying form of abuse. Thus, when methods of pharmacovigilance are used to evaluate particular forms of addictive behaviours, the substance-related risk for different forms of abuse (recreational use, inducing a “high”, a classical form of dependence with tolerance and withdrawal, or PNE) may be assessed. However, concerning substances that are known to be used for PNE, there is heterogeneity regarding the presence of abuse outside the field of PNE. Methylphenidate is the only substance used for PNE that is known to feature an abuse potential outside the field of PNE (e.g., recreational use, to induce weight loss or euphoric effects) [24] and may cause dependence, particularly when administered parenterally [25]. The other substances used for PNE do not feature a known potential for abuse and dependence outside the context of PNE. It was shown in clinical studies that atomoxetine in comparison to methylphenidate does not feature abuse liability [26,27]. Modafinil was also evaluated to have no abuse potential in the sense of recreational use or application to induce euphoric feelings [28,29]. Outside the field of PNE, abuse or dependence related to acetylcholine esterase inhibitors or memantine is completely unknown.

In this study, we tested if quantitative signal detection may be sensitive to detecting an increased risk for PNE related to agents known to be used for PNE. Therefore, we evaluated the risk for abuse and dependence of prescription agents that are known to be used for PNE (methylphenidate, atomoxetine, modafinil, acetylcholine esterase inhibitors, and memantine). OTC drugs and other psychoactive agents (caffeine, illicit drugs) were not considered. We hypothesized that the method of quantitative signal detection—which is insensitive in regards to the form of abuse (recreational use, induction of euphoric feelings, a classical form of dependence with tolerance and withdrawal, or PNE) that underlies a particular addictive behaviour and is the subject of an ADR report—will consistently indicate an increased risk for abuse and dependence related to methylphenidate (which is known to also be abused outside the field of PNE) and may (sporadically) also reveal an increased risk for abuse and dependence related to agents that do not have a known abuse potential outside the field of PNE (this is true for all agents except for methylphenidate). Verification of this hypothesis could refer to possible sensitivity of quantitative signal detection in regards to PNE performed with agents that do not feature an abuse potential outside the field of PNE.

## 2. Results

Table 1 displays the numbers of cases (ADR reports related to a particular agents and DAAD) per dataset and the number of all substance-related ADRs per dataset without SMQ restriction. Both control substances (diazepam and lorazepam) were statistically significantly associated with DAAD in the total dataset and all analyzed country-specific datasets (see Table 2). Several country-specific datasets did not allow for calculating RORs related to several substances due to missing reports on ADRs related to DAAD or the substance of interest. Regarding agents used for PNE methylphenidate was associated with DAAD in all analyzed datasets (except Italy: no appropriate reports were documented). Modafinil was associated with DAAD in the total dataset (ROR, 2.7 (95% CI, 2.2–3.3)), Germany (ROR, 4.6 (1.8–11.5)), and USA (ROR, 2.0 (95% CI, 1.6–2.5)). Atomoxetine was associated with DAAD in the total dataset (ROR, 1.3 (95% CI, 1.2–1.5)) and in the UK (ROR, 3.3 (95% CI, 1.8–6.1)). Apart from memantine, which was associated with DAAD in Germany (ROR, 1.8 (95% CI, 1.0–3.2)), no other agents of the group of antidementia drugs were associated with DAAD.

## 3. Discussion

PNE should be regarded as a form of abuse and is thus a potential subject to pharmacovigilance and “addictovigilance”, respectively [19]. Addictive behaviours related to pharmaceutical agents are possible ADRs, and, in this respect, may be reported to spontaneous reporting systems [20]. Methods of pharmacovigilance such as disproportionality analysis have been repeatedly used with success to identify the risk for abuse and dependence of pharmaceutical agents [19,21,22]. In the present study, we calculated RORs to evaluate the potential for abuse and dependence of prescription drugs known to be used for PNE in order to test if quantitative signal detection is sensitive to detecting agents used for PNE. We hypothesized that the method of quantitative signal detection—which is insensitive in regards to the underlying form of abuse—will consistently indicate an increased risk for DAAD related to substances with a known abuse potential outside the field of PNE (methylphenidate) and may also reveal an increased risk for abuse/dependence related to agents that do not have a known abuse potential outside the field of PNE (all included substances other than methylphenidate).

In all datasets, we consistently found a statistical association of both control substances (lorazepam and diazepam) with DAAD, indicating that the used method of quantitative signal detection is generally suitable for detecting substances with a potential to induce addictive behaviours. However, our hypothesis was only partly verified by the results. As expected, methylphenidate was associated with DAAD in all analyzed datasets (except Italy, where no appropriate reports were present). An increased risk for DAAD was also found for atomoxetine (total dataset, Australia, and UK), memantine (Germany), and modafinil (total dataset, Germany, and USA). Memantine was only associated with DAAD in Germany and not in the total dataset, where larger numbers of cases were evaluated and increased sensitivity was present. Thus, this finding should be interpreted with caution. No other substance of the group of antidementia drugs (acetylcholine esterase inhibitors) was statistically associated with DAAD. Although not precisely studied, antidementia drugs were evaluated to be less frequently used for PNE [3]. This may explain the absence of positive associations between DAAD and antidementia drugs in our study. As atomoxetine and modafinil do not feature a known abuse potential outside the field of PNE, it is likely that the positive associations between DAAD and these substances found in our analysis are due to PNE, even though the involvement of forms of abuse other than PNE cannot be ruled out. The substance-specific RORs calculated based on the total dataset indicate that only atomoxetine, methylphenidate, and modafinil are associated with an increased risk of abuse. This may be due to the assessment that these three substances are most frequently used for PNE [3].

As the practice of PNE is primarily found in Western countries, we have only analyzed drug safety data related to Western countries with important pharmaceutical markets. The extent of the analyzed datasets, however, featured a considerable variance between the evaluated countries. There were, for example, no reports related to methylphenidate and DAAD in Italy, although this substance is approved in Italy and methylphenidate-related addictive behaviours are also likely to be present in this country. In several other country-specific datasets (particularly Australia, Canada, France, and Spain), neither substance- nor SMQ-specific ADR reports were present. This strongly suggests that underreporting the phenomenon of incorrectly low reporting rates of ADR [30] is present in the evaluated datasets. Naturally, underreporting affects not only the practice of PNE but every form of addictive behaviour related to a pharmaceutical agent. Thus, the absent associations (either ROR < 1 or absent appropriate ADR reports) between particular substances and DAAD found in our study must not simply be interpreted as absent substance-specific abuse potential (although this interpretation is most likely); the absent associations may also be an expression of insensitivity of the used method due to underreporting.

As the applied database query strategy and the used method of quantitative signal detection do not allow consideration of case-sensitive data (e.g., age, sex, comorbid somatic and/or mental disorders), factors that may increase the risk for abuse and/or dependence related to the evaluated substances could not be included. This circumstance is a major limitation of our study as the abuse of alcohol, caffeine and nicotine is known to alter dopamine transmission in limbic and frontal brain areas that are involved in the development of substance use disorders [31,32].

## 4. Materials and Methods

### 4.1. Database

Following thalidomide-related events, the World Health Organization (WHO) established the International Drug Monitoring Programme in 1968 in order to improve the drug safety of marketed drugs. Currently (as of May 2016), the national pharmacovigilance centres (NPC) of 124 countries take part in this programme. Furthermore, the NPCs of 29 countries are associated members (for further details, available on: http://www.umc.org). NPCs report ADR to a central institution, the Uppsala Monitoring Centre (UMC) in Sweden. Recording of ADR data at the UMC has been performed since 1968. The drug safety data contained in ADR reports submitted to the UMC are systematically evaluated and recorded in a particular pharmacovigilance database that is called VigiBase™. VigiBase™ is the worldwide largest pharmacovigilance database (for further details, available on: http://ww.umc-products.com). The ADR reports that are submitted to the UMC originate from variable sources such as regulatory authorities and voluntary sources (e.g., patients and health professionals) as well as from the pharmaceutical industry. These differences are the result of country-specific differences concerning the pharmacovigilance system and especially the spontaneous reporting systems of the respective country. It has to be considered that a causality assessment, which is the assessment of the probability that the respective drug has factually caused the reported adverse event, is not systematically performed by every participating NPC. Most importantly, it has to be taken into account that drug safety data represented in VigiBase™ and their possible implications do not represent the opinion of the WHO.

### 4.2. Database Query and Search Strategy

VigiBase™ was accessed in April 2016 (index date) by a preceding online application at the WHO. Data were obtained by using the standardised Medical Dictionary for Regulatory Activities (MeDRA). MedDRA is a validated medical terminology thesaurus that is internationally used by regulatory authorities [33]. With the aim of providing a very specific and standardised medical terminology that facilitates international sharing of regulatory information for medical products used by humans, it was created in the 1990s by the International Conference on Harmonisation of Technical Requirements for Registration of Pharmaceuticals for Human Use (ICH) (available on: http://www.meddra.org). The glossary of the MedDRA is organized in a hierarchical structure and covers about 66,000 terms at the lowest level. There are five levels of this hierarchical structure, and each level is characterized by a particular granularity: System Organ Class (SOC), High Level Group Term (HLGT), High Level Term (HLT), Preferred Term (PT), and Lowest Level Term (LLT). For database queries, a specific tool exists that uses groupings of terms that relate to a defined medical condition or area of interest; these groupings of terms are the Standardized MedDRA Queries (SMQ). Particular SMQs are further specified in broad (increased sensitivity) and narrow (increased specificity) scope grouping of relevant terms. For the present study, MedDRA 19.0 (available on: http://www.meddra.org/) was used.

For the present study, the SMQ-term “Drug abuse and dependence” (DAAD) was used; it includes the use of medication not needed for therapeutic use and excludes events associated with withdrawal and intoxications and medication error/maladministration. In order to facilitate maximum precision (increased specificity), we retrieved data for ADR reports related to the narrow scope of the SMQ DAAD (thus excluding drug tolerance, drug level about therapeutics, overdoses, substance-induced mood-disorders, etc.). Data retrieved from the VigiBase™ database cover the period 1968–2016 (April 2016). Data were retrieved for the following substances: atomoxetine, donepezil, galantamine, memantine, methylphenidate, modafinil, and rivastigmine. Due to their known abuse and dependence potential, ADR data for diazepam and lorazepam were retrieved as positive controls. Numbers of all ADR reports (no restriction by SMQs) and numbers of ADR reports related to the SMQ “DAAD” were retrieved for each substance and in total (also for each substance) as recorded at the index date. The included ADR reports were not evaluated for causality, implicating that all levels of causality were included. Statistical analysis was done for each substance separately using the total dataset and country-specific datasets (here, subsets of the respective country as recorded in VigiBase™ were evaluated). Evaluation of country-specific data was done only for Western countries with important pharmaceutical markets based on data from the IMS World Review Analyst 2014 (for further details, available on: www.imshealth.com/portal/site/imshealth). The included countries were: Germany, Canada, France, Australia, Italy, Germany, Spain, USA, and UK.

No separate evaluation of the plausibility of the included ADR reports was performed as all ADR reports undergo rigorous systematic screenings before definite recording in VigiBase™. The described type of database query does not allow for obtaining case-sensitive information (e.g., age, gender, co-medication, substance use disorders, particularly alcohol, nicotine and caffeine abuse and/or dependence, etc.).

### 4.3. Statistical Analysis

In the science of pharmacovigilance, a signal (information that arises from one or more multiple sources including observations or experiments), which suggests a new potentially causal association, or a new aspect of a known association between an intervention and an event or set of related events, either adverse or beneficial, that is judged to be of sufficient likelihood to justify verificatory action” (“Practical Aspects of Signal Detection in Pharmacovigilance”, Report of the Council for International Organizations of Medical Sciences (CIOMS) Working Group VIII, Geneva 2010) is defined as an indication for a possible causal relation between an adverse event and the use of a pharmaceutical agent; the detection of signals is of paramount importance for the drug safety evaluation of drugs in the post-marketing setting [34,35,36]. In quantitative signal detection, data mining methods are frequently used and applied to databases of spontaneous reports of ADR [23]. Based upon differences from the background frequency, a highly disproportionate representation of a drug/adverse event association may refer to an important safety signal [37,38]. In our study, we used a case/non-case methodology and calculated reporting odds ratios (RORs) as a measure for disproportionality [38,39] to assess the strength of the statistical association between treatment with a particular drug (see above: included agents) and the occurrence of the event DAAD. RORs were calculated with a 95% confidence interval (CI) [39,40] and based on a two-by-two contingency table (ROR = (a/c)/(b/d) = ad/bc). The mathematical formula for the 95% CI interval was: $e^{\ln\left(\mathrm{ROR}\right)\;\pm \;1.96\;\sqrt{\left(1/a\;+\;1/b\;+\;1/c\;+\;1/d\right)}}$ [36]. Cases were defined as ADR reports related to DAAD and non-cases as ADR reports other than DAAD. “Exposure” was defined as nomination of any of the abovementioned drugs in an ADR report.

## 5. Conclusions

As demonstrated in our analysis of ADR data related to methylphenidate, diazepam and lorazepam, quantitative signal detection is suitable for detecting substances with a risk for abuse and dependence. However, its sensitivity regarding PNE is limited. Atomoxetine and modafinil were associated with an increased risk for abuse in several datasets, although an abuse potential outside the field of PNE is unknown. This may indicate that quantitative signal detection is sensitive regarding PNE in these cases, even though other explanations as forms of abuse other than PNE cannot be ruled out. Other substances known to be used for PNE (antidementia drugs) were not associated with an increased risk for abuse, either due to their presumably low use for PNE compared with other substances or due to underreporting.

## Figures and Tables

**Table 1:** Numbers of ADR reports related to SMQ “Drug abuse and dependence” (narrow scope) (~cases) and total numbers of ADR reports per drug per dataset in VigiBase™.

Cases/All ADR-Reports per Drug per Dataset (No SMQ Restriction)									
Substance	Total Dataset	Australia	Canada	France	Germany	Italy	Spain	United Kingdom	USA
Atomoxetine	248/21,265	1/148	5/406	0/7	7/496	0/119	0/133	10/776	221/17,178
Donepezil	42/11,294	1/377	0/574	0/875	4/528	2/386	0/266	1/1266	33/3567
Galantamine	9/3927	0/152	0/280	0/382	5/341	0/60	0/111	1/314	3/1008
Memantine	40/6993	0/45	0/74	0/580	12/295	0/213	0/150	0/249	27/4534
Methylphenidate	728/28,591	11/295	44/1472	42/408	133/1472	0/123	11/326	16/1170	340/16,989
Modafinil	85/3586	0/28	0/72	1/154	5/51	0/7	0/16	2/170	75/2913
Rivastigmine	10/11,117	0/191	0/1426	0/725	0/698	0/468	0/314	0/522	8/3048
**Positive Control Substances**									
Diazepam	4359/23,119	38/1018	118/1060	80/1251	531/1673	264/592	23/476	94/1062	2887/11,291
Lorazepam	3251/19,966	1/146	60/680	57/1489	1763/3052	417/1136	26/812	36/460	662/8396

ADR = adverse drug reaction; SMQ = Standardised Medical Dictionary for Regulatory Activities query; USA = United States of America.

**Table 2:** Reporting odds ratios (RORs) related to SMQ “Drug abuse and dependence” (narrow scope) *.

ROR (95% CI)									
Substance	Total Dataset	Australia	Canada	France	Germany	Italy	Spain	United Kingdom	USA
Atomoxetine	1.3 (1.2–1.5)	2.2 (0.3–15.7)	1.4 (0.6–3.3)	-	0.6 (0.3–1.3)	-	-	3.3 (1.8–6.1)	1.0 (0.9–1.1)
Donepezil	0.4 (0.3–0.6)	0.9 (0.1–6.1)	-	-	0.3 (0.1–0.9)	0.5 (0.1–2.2)	-	0.2 (0.0–1.4)	0.7 (0.5–1.0)
Galantamine	0.3 (0.1–0.5)	-	-	-	0.6 (0.3–1.5)	-	-	0.8 (0.1–5.7)	0.2 (0.1–0.7)
Memantine	0.6 (0.5–0.9)	-	-	-	1.8 (1.0–3.2)	-	-	-	0.5 (0.3–0.7)
Methylphenidate	2.9 (2.7–3.1)	12.6 (6.9–23.4)	3.4 (2.5–4.5)	22.6 (16.4–31.2)	4.2 (3.5–5.0)	-	23.6 (12.8–43.5)	3.5 (2.1–5.7)	1.5 (1.4–1.7)
Modafinil	2.7 (2.2–3.3)	-	-	1.3 (0.2–9.1)	4.6 (1.8–11.5)	-	-	3.0 (0.7–12.0)	2.0 (1.6–2.5)
Rivastigmine	0.1 (0.05–0.2)	-	-	-	-	-	-	-	0.2 (0.1–0.4)
**Positive Control Substances**									
Diazepam	26.5 (25.7–27.4)	12.9 (9.3–18.0)	14.0 (11.5–17.0)	13.6 (10.9–17.2)	20.4 (18.4–22.7)	91.0 (77.0–107.4)	35.5 (23.0–54.6)	25.0 (20.2–31.0)	26.5 (25.4–27.6)
Lorazepam	22.0 (21.2–22.9)	2.2 (0.3–15.9)	10.6 (8.1–13.9)	7.9 (6.0–10.3)	67.1 (62.3–72.3)	69.5 (61.3–79.0)	23.3 (15.5–34.8)	21.4 (15.2–30.2)	6.4 (5.9–7.0)

CI = confidence interval; ROR = reporting odds ratio; SMQ = Standardised Medical Dictionary for Regulatory Activities Query; - = calculation of ROR not possible due to missing ADR reports related to the used SMQ or the particular substance; USA = United States of America; * = calculations of ROR based on *n* = 12,784,357 ADR reports in total and *n* = 115,145 ADR reports related to SMQ “Drug abuse and dependence” (narrow scope) at the index date recorded in Vigibase™ (all ADR reports per country: Australia, *n* = 316,854; Canada, *n* = 395,046; France, *n* = 531,828; Germany, *n* = 527,904; Italy, *n* = 297,005; Spain, *n* = 255,738; United Kingdom, *n* = 688,900; USA, *n* = 6,288,366).

## References

[1] Franke A., Northoff R., Hildt E. (2015). The case of pharmacological neuroenhancement: Medical, judicial and ethical aspects from a german perspective. Pharmacopsychiatry.

[2] Ragan C., Bard I., Singh I. (2013). What should we do about student drug use of cognitive enhancers? An analysis of current evidence. Neuropharmacology.

[3] Franke A., Bagusat C., Rust S., Engel A., Lieb K. (2014). Substances used and prevalence rates of pharmacological cognitive enhancement among healthy subjects. Eur. Arch. Psychiatry Clin. Neurosci..

[4] Micoulaud-Franchi J., MacGregor A., Fond G. (2014). A preliminary study on cognitive enhancer consumption behaviors an motives of French Medicine and Pharmacology students. Eur. Rev. Pharmacol. Sci..

[5] Ott R., Biller-Andorno N. (2014). Neuroenhancement among Swiss students—A comparison of users and non-users. Pharmacopsychiatry.

[6] Maher B. (2008). Poll results: Look who’s doping. Nature.

[7] Dietz P., Soyka M., Franke A. (2016). Pharmacological neuroenhancement in the field of economics—Poll results from an online survey. Front. Psychol..

[8] Heinz A., Kipke R., Heimann H., Wiesing U. (2012). Cognitive euroenhancement: False asumptions in the ethical debate. J. Med. Eth..

[9] Faber N., Savulescu J., Douglas T. (2016). Why is cognitive enhancement deemed unacceptable? The role of fairnes, deservingness, and hollow achievements. Front. Psychol..

[10] Franke A., Soyka M. (2015). Pharmacological cognitive enhancement from a perspective of misuse and addiction. Fortschr. Neurol. Psychiatry.

[11] Vargo E., Petróczi A. (2016). “It was me on a good day”: Exploring the smart drug use phenomenon in England. Front. Psychol..

[12] Clemow D., Walker D. (2014). The potential for misuse and abuse medications in ADHS: A review. Postgrad. Med..

[13] Battleday R., Brem A. (2015). Modafinil for cognitive neuroenhancement in healthy non-sleep-deprived subjects: A systematic review. Eur. Neuropsychopharmacol..

[14] Repantis D., Schlattmann P., Laisney O., Heuser I. (2010). Modafinil and methylphenidate for neuroenhancement in healthy individuals: A systematic review. Pharmacol. Res..

[15] Repantis D., Laisney O., Heuser I. (2010). Acetylchlinesterase inhibitors and memantine for neuroenhancement in health individuals: A systematic review. Pharmacol. Res..

[16] Graf H., Abler B., Freudenmann R., Beschoner P., Schaeffeler E., Spitzer M., Schwab M., Grön G. (2011). Neural correlates of error monitoring modulated by atomoxetine in healthy volunteers. Biol. Psychiatry.

[17] Hildt E., Lieb K., Bagusat C., Franke A. (2015). Reflections on addiction in students using stimulants for neuroenhancement: A preliminary interview study. BioMed Res. Int..

[18] Micallef J., Lapeyre-Mestre M. (2015). Addictovigilance: The pharmacological challenge of assessment and prevention of risky substances. Therapie.

[19] Lapeyre-Mestre M., Dupui M. (2015). Drug abuse monitoring: Which pharmacoepidemiological resources at the European level?. Therapie.

[20] Goldman S. (1998). Limitations and strengths of spontaneous reports data. Clin. Ther..

[21] Bossard J., Ponté C., Dupouy J., Lapeyre-Mestre M., Jouanjus E. (2016). Disproportionality analysis for the assessment of abuse and dependence potential of pregabalin in the French Pharmacovigilance Database. Clin. Drug Investig..

[22] Cossmann M., Kohnen C., Langford R., McCartney C. (1997). Tolerance and drug safety of tramadol use. Results of international studies and data from drug surveillance. Drugs.

[23] Bate A., Evans S. (2009). Quantitative signal detection using spontaneous ADR reporting. Pharmacoepidemiol. Drug Saf..

[24] Bogle K., Smit B. (2009). Illicit methlphenidate use: A review of prevalence, availability, pharmacology, and consequences. Curr. Drug Abuse. Rev..

[25] Volkow N., Swanson J. (2003). Variables that affect the clinical use and abuse of methylphenidate in the treatment of ADHD. Am. J. Psychiatry.

[26] Heil S.H., Holmes H.W., Bickel W.K., Higgins S.T., Badger G.J., Laws H.F., Faries D.E. (2002). Comparison of the subjective, physiological, and psychomotor effects of atomoxetine and methylphenidate in light drug users. Drug Alcohol. Depend..

[27] Upadhyaya H.P., Desaiah D., Schuh K.J., Bymaster F.P., Kallman M.J., Clarke D.O., Durell T.M., Trzepacz P.T., Calligaro D.O., Nisenbaum E.S. (2013). A review of the abuse potential assessment of atomoxetine: A non-stimulant medication for attention-deficit/hyperactivity disorder. Psychopharmacology.

[28] Jasinski D. (2000). An evaluation of the abuse potential of modafinil using methylphenidate as a reference. J. Psychopharmacol..

[29] Jasinski D., Kovacevic-Ristanovic R. (2000). Evaluation of the abuse liability of modafinil and other drugs for excessive daytime sleepiness associated with narcolepsy. Clin. Neuropharmacol..

[30] Martin R., Kapoor K., Wilton L., Mann R. (1998). Underreporting of suspected adverse drug reactions to newly marketed (“black triangle”) drugs in general practice: Observational study. BMJ.

[31] Doyon W., Thomas A., Ostroumov A., Dong Y., Dani J. (2013). Potential substrates for nicotine and alcohol interactions: A focus on the mesocorticolimbic dopamine system. Biochem. Pharmacol..

[32] Cauli O., Morelli M. (2005). Role of dopamine in the behavioural actions of nicotine related to addiction. Eur. J. Pharmacol..

[33] Brown E., Wood L., Wood S. (1999). The medical dictionary for regulatory activities (MedDRA). Drug Saf..

[34] Van Manen R., Fram D., DuMouchel W. (2007). Signal detection methodologies to support effective safety management. Expert Opin. Drug Saf..

[35] Gould A. (2003). Practical pharmacovigilance analysis strategies. Pharmacoepidemiol. Drug Saf..

[36] Ooba N., Kubota K. (2010). Selected control events and reporting odds ratio in signal detection methodology. Pharmacoepidemiol. Drug Saf..

[37] Finney D. (1971). Statistical logic in the monitoring of reactions to therapeutic drugs. Methods Inf. Med..

[38] Van Puijenbroek E.P., Bate A., Leufkens H.G., Lindquist M., Orre R., Egberts A.C. (2002). A comparison of measures of disproportionality for signal detection in spontaneous reporting systems for adverse drug reactions. Pharmacoepidemiol. Drug Saf..

[39] Rothman K., Lanes S., Sacks S. (2004). The reporting odds ratio and its advantages over the proportional reporting odds ratio. Drug Saf..

[40] Evans S. (2000). Pharmacovigilance: A science or fielding emergencies?. Stat. Med..

